# Supplementary material for: Are carbon emissions trading and green financial instruments synergistic? -Comprehensive quantitative research based on content analysis
Source: PLoS One. 2024 Mar 7;19(3):e0298601. doi: 10.1371/journal.pone.0298601 (PMC10919601; doi:10.1371/journal.pone.0298601)
Supplement: S1 Data — (ZIP) [file pone.0298601.s001.zip › Data/地级市Z_F工作报告.zip!/╣π╢1⁄2/╓Θ║ú╩╨/└┤╫╘╣1⁄2╓┌║┼í╛┬φ┐╦╩2╛▌═°í┐_2014.docx]

各位代表：

　　现在，我代表市人民政府向大会报告工作，请代表们审议，并请市政协委员和其他列席人员提出意见。

　　一、2013年工作回顾

　　过去一年，我市有效应对错综复杂的国内外经济形势，坚持稳中求进，统筹稳增长、调结构、促改革和惠民生，经济发展稳中向好，转型升级扎实推进，改革开放继续深化，民生保障持续改善，较好地完成了市八届人大三次会议确定的主要目标任务。

　　（一）加快转型升级，经济提质增效。

　　经济增长稳中提速。我们多措并举，有效促进了经济逐季回升、一路向好。预计实现地区生产总值1651亿元，增长10.3%，高于全省增速1.8个百分点，人均GDP约1.6万美元，超额实现了增长目标。城镇居民人均可支配收入36375元，增长10.3%；农村居民人均纯收入15140元，增长13%，均超过国内生产总值增幅。固定资产投资总额961亿元，增长23%，增速连续三年位居珠三角第一。进出口总额541.7亿美元，增长18.6%，比上一年大幅提高了30.2个百分点。社会消费品零售总额717亿元，增长13%。公共财政预算收入194.2亿元，增长19.4%。全市本外币存贷款余额分别为4121.6亿元和2071.9亿元，增长19.5%和7.9%。居民消费价格涨幅控制在2.3%。

　　产业素质整体提高。产业结构优化升级，“三高一特”产业发展形成新动力。工业发展冲出低谷，规模以上工业企业实现增加值738亿元，增长11.2%，企业利润总额增长30%。先进制造业、高技术制造业增加值占规模以上工业增加值比重分别达到44%和24.2%。规模以上高新技术工业企业增加值增长14.2%，高于工业增速3个百分点。现代服务业增加值增长10.9%，占服务业比重提升到57%，金融业增加值占GDP比重提升到5.7%。大型骨干企业规模增大，年营业收入超10亿元的企业预计达80家，其中超百亿元企业5家，格力电器实现年营业总收入1200亿元。民营经济占GDP比重提升到32%。海洋产业产值突破700亿元，增长27%。斗门生态农业园成为国家农业科技园区和全国首个河口渔业示范区。外经贸转型提质，加工贸易委托设计和自主品牌混合生产方式出口占比增至60%，一般贸易进出口增长约30%。实际利用外资16.9亿美元，增长16.6%，72个新项目签约落户，总投资445亿元。

　　创新驱动能力增强。全社会研发投入占GDP比重达2.52%，每万人口拥有研发人员和每百万人口年发明专利申请量居全省第二，连续七次获评全国科技进步先进市。新增国家高新技术企业63家，科技型企业规模扩大。省、市级工程中心和企业技术中心总数达到287家，研发能力提高。实施《人才开发促进条例》，引进3D打印、中星微电子等一批顶尖创新团队和领军人才，一大批海外高层次留学人员到珠海干事创业，高素质人才队伍集聚发展。

　　（二）加快重大平台开发建设，多极增长支撑力不断提升。

　　横琴新区开发跨越前进。自贸区申报取得积极进展，产业发展指导目录、进口税收等政策实施细则出台。现代服务产业高地快速崛起，累计引进世界500强企业38家，总投资2400亿元的61个大项目扎实推进，十字门中央商务区建设进展顺利，长隆国际海洋度假区首期即将开业、企鹅酒店等三大新项目动工。成功举办第一届中国国际马戏节，展现了横琴的国际影响力。金融创新发展，已落户金融类企业和机构321家，第一家外资银行代表处——澳门国际银行代表处获批，跨境人民币结算业务快速扩大，广东金融资产交易中心和珠海产权交易中心开业。岛内主干路网基本建成，横琴岛澳门大学新校区投入使用，二线通道和环岛监控项目竣工，已可封关运作。

　　多极增长带动能力增强。高栏港区跨入“亿吨大港”，吞吐量增长29.3%，港口货物吞吐能力接近1.5亿吨。新建成产业项目16个，新引进项目13个、总投资超过220亿元，先进制造业扩量增效。高新区迈入二次振兴新时期，GDP突破百亿，规模以上工业增加值增速全市居首，“一区五园”工业总产值占全市比重升至49%，实现了争先进位。特色园区竞相发展，航空产业园建设提速，中航通飞产业基地多款飞机研发制造顺利推进，西锐SR2X飞机总装生产线安装完成，利捷公务航空等一批新项目落户。富山工业园临港制造业、滨海医药港、环保产业园等快速发展，修正药业等新项目进驻，惠普智慧产业园、中国北车珠海基地等项目加快建设。保税区加快向商贸服务转型，进口酒类交易中心和珠澳跨境区进口街加快发展。海洋海岛开发有序推进，“蓝色万山、海洋强区”建设取得新进展。

　　（三）加快完善交通基础设施，交通枢纽建设掀起新热潮。

　　进一步完善交通枢纽功能，启动了交通建设“三年大会战”，规划新建一批外通内连的关键性工程。港珠澳大桥主体工程和珠海连接线加快建设，人工岛造成，口岸工程开工。高栏高速和机场高速已全线建成通车，疏港铁路专用线一期主体工程、珠海大道全线和迎宾南路等一批道路改扩建完工。有轨电车1号线、金琴高速主线等工程开工，金湾互通立交、井岸二桥、横琴二桥等一批项目快速推进。高栏港基础设施建设保持强劲势头，10万吨级进港主航道建成运行，15万吨级主航道扩建动工，15万吨级干散货码头等泊位对外开放，新增港口吞吐能力超过3000万吨。珠海机场首飞国际航空货运包机，年旅客吞吐量达290万人次，增长近4成。拱北口岸新扩建联检楼首层通关大厅启用，通关环境明显改善。绿色交通加快发展，新投放环保公交车和出租车550多台、公共自行车5000辆，市民出行更加便捷。

　　（四）统筹区域协调发展，生态宜居城市建设水平持续提高。

　　新城开发高起点推进。新编城市概念性空间规划，修订了城市总体规划，高标准推进新一轮城市开发建设。西部生态新城首期开发区域建设全面启动。金湾航空城完成核心区土地清理核实工作。平沙新城市政配套加快完善。唐家湾科教新城完成唐家湾地区空间整体设计，高新区北围、TOD市政道路暨产业启动区开工。富山产业新城完成总体战略、空间布局等规划设计，雷蛛片区水利基础设施填土工程加快建设。

　　美丽珠海行动全面实施。全力创建全国文明城市和生态文明示范市，实施《生态文明建设促进条例》，推进美丽珠海行动五年计划，新开展一批环境宜居重大项目。全面实施“天更蓝、水更清、城更美、环境更安全”四大重点工程，改造了49条道路的绿化景观，新建了65公里绿道、96公里生态景观林带、2.6万亩碳汇林。完成节能减排约束性指标，淘汰营运“黄标车”7000多辆，二氧化硫排放量预计削减16.53%，单位GDP能耗居全省第三低位。华新生态环保园生活垃圾处理环保工厂开工建设，中信生态环保产业园首期项目完成征地。城镇生活垃圾无害化处理率达到100%，污水处理率超过87%。整治违法用地2388亩、违法建筑260多万平方米，土地承载力进一步提高。完善城市更新规划，拱北口岸片区、城市之心、九洲动感活力轴、“三溪”人居环境改善工程等49个项目扎实推进。海滨泳场改造提升一期工程完成。首个有轨电车TOD小镇开工建设。77家农贸市场改造升级，方便了广大市民。

　　幸福村居创建走在全省前列。大力推进15个示范村318个项目建设，特色产业发展、宜居环境提升等六个方面工作成效显著，成为深受农民欢迎的民心工程。农村综合改革不断深化，三个行政区全部建成“三资”交易管理平台，斗门区获得“广东治理创新奖”中唯一的“农村改革奖”。

　　智慧城市建设扎实有效。实施智慧城市建设总体规划和三年行动计划，开展国家智慧城市试点创建工作，新启动了一批智慧项目。一批电子泊车系统和电子站牌等智能交通设施建成使用。推动光纤入户，互联网普及率达到80.4%。网上办事大厅实现省、市、区、镇四级联通，100%的部门和事项入驻，98%的行政审批事项可在网上办理。市民网页和企业网页、12345市民服务热线开通运行。

　　区域交流合作成果扩大。珠港澳合作机制进一步完善，珠澳共建天气雷达项目试运行，粤澳新通道项目批准建设。珠中江三市新签海洋经济区域合作、传染病联防联控等8项合作协议，编制完成三地城市空间协调发展规划，前山河流域环境共治等29个项目加快实施。圆满完成揭阳三县（市）扶贫开发“双到”和揭阳、茂名两个产业转移园合作共建任务。新一轮对口茂名、阳江扶贫开发“双到”工作开局良好。

　　（五）着力加强社会建设，民生保障继续加强。

　　民生财政支出有新投入。全市公共财政预算用于九项民生支出153.1亿元，占支出总额61.7%，办成了一批人民群众看得见、得实惠的好事实事。

　　民生保障水平有新提高。促进更高质量就业，城镇新增就业4.33万人，登记失业率控制在2.28%以内。企业最低工资标准提高到1380元，增长20%。实施大病补充医疗保险，职工医保和城乡居民医保实际报销比例最高达到94%和81%，报销额度最高可达62万元和40万元。新农保和城居保月人均养老金分别增长66%和38%，待遇保障水平位居全省前列。城镇和农村居民最低生活保障标准提高了20%以上。为1.1万名残疾人发放基本生活津贴1920多万元。率先实现所有流动就业人员医疗保险关系转移接续，异地务工人员纳入生育保障范围，1800人通过积分入户，义务教育阶段免费教育补贴覆盖面扩大到具有珠海学籍的全部非户籍学生。超额完成省下达的住房保障目标任务，新开工保障房3219套、基本建成5944套，发放廉租房补贴271.3万元，大镜山保障房分配入住。

　　社会事业发展有新进步。公办义务教育标准化学校实现全覆盖，学前教育三年行动计划圆满完成，新建10所镇中心幼儿园全面推进，市十一中等一批教育设施建成使用。启动公立医院改革，实施公立医院与社区卫生服务机构“联建协管”、医师多点执业等机制，稳步推进区域医疗“一卡通”。连续第25年完成人口计生目标任务，人口整体素质有新提高。文体事业加快发展，国有文化资产监管新体系建立，特区报业、广播影视两大传媒集团组建工作启动。珠海文化公园完成改造，市文化馆新馆竣工，珠海大剧院、规划展览馆和博物馆主体结构封顶。农家（社区）书屋、广播、电视实现全覆盖，每万人公共文化设施面积高于全省平均水平300平方米。一大批社区体育公园建成投用。我市体育健儿参加第十二届全运会取得史上最好成绩。成功举办国际半程马拉松赛、国际沙滩音乐节等城市文体旅游品牌活动。

　　平安珠海建设有新成效。强化社会矛盾纠纷排查调处和信访维稳工作，加强社会治安综合治理，新推40个“全民创安”重点项目，社会治安良好。安全生产形势稳定，亿元GDP生产安全事故死亡率下降16.6%。应急体系进一步完善，预防和处置突发事件能力提高。国家食品药品安全示范市创建工作全面启动，基层监管体系加快完善。国家安全、司法行政、海防打私、民族宗教、双拥优抚等工作进一步加强。

　　（六）推进改革创新，经济社会发展活力动力增强。

　　经济体制改革成效明显。率先改革商事登记制度，新登记企业、注册资本金同比分别增长57%和208.7%。深入开展“两建”工作，开通政府公共联合征信平台和珠海信用网，商业信用环境指数在全国排名前列。设立创业投资引导基金，“营改增”实施以来累计减税6.23亿元。充分发挥市属国有企业的支柱性作用，新组建了农业投资控股集团，市管企业资产总额、国有权益分别增长27.4%和26%，承担了交通建设、产业发展、新区开发等重点项目41个、总投资1180多亿元。

　　社会体制改革力度加大。推动出台《社会建设条例》，成为全国首个远程社会工作培训试点城市，社会组织培育发展中心投入使用，每万人拥有社会组织数达到13.4家，注册志愿者达到20.3万人。完善城乡基层社会治理，建立行政事务社区准入机制，90.2%的行政村建立了村务监督委员会制度。

　　行政体制改革不断深入。完成第五轮行政审批制度改革，减少审批事项129项，压缩47.6%，提前两年完成省下达的改革目标。建立了“集装箱式”并联审批机制，审批效能进一步提高。理顺政务服务与公共资源交易管理体制，启动市会议展览局等六家单位法定机构试点。开展了行政复议委员会试点。开放市民监督平台，建立首个政府投资工程廉情评比指标体系。深入开展“转作风、提效能”活动，严格落实中央“八项规定”，全市性会议、发文和“三公”经费分别减少32.6%、44.2%和14%，公开了第一批46个单位的经费预算。认真办理人大议案3件、代表建议197件、政协提案433件，办理质量有新提高。

　　各位代表，过去一年取得的新发展、新成就，使我们的城市更加美丽、生活更加美好，也让我们倍感自豪、倍增自信。成绩来之不易，归功于全市人民的共同努力、团结奋进。在这里，我代表市人民政府，向各位代表、政协委员和全市人民表示衷心感谢！

　　但是，我们也清醒认识到，经济社会发展中仍然存在一些老问题和新矛盾：一是距离进入珠三角经济第二梯队还有较大差距，经济下行压力仍然存在，调结构转方式、促进经济提速提质发展的任务还很艰巨。二是城市功能还需要提升完善，基础设施水平还不适应现代化城市发展，市政建设、交通发展和园区配套还要加快推进，城市安全和管理水平有待提高。三是城乡区域发展不平衡、不协调问题仍然突出，基本公共服务均等化水平还要大力提升。四是政府职能转变还没有完全到位，加快改革创新的任务迫切而繁重。对此，我们将高度重视、认真解决。

　　二、2014年工作安排

　　综观当前国内外发展环境，世界经济仍处于深度调整期，维持缓慢复苏态势，我国经济长期向好的基本面没有改变，全面深化改革将释放经济增长新动力，保持经济持续健康发展具有较好基础。但国际金融危机影响具有长期性，国内经济下行压力仍然较大。综合判断我市今年发展局势，机遇和挑战并存，总体上还处在可以大有作为的重要战略机遇期。我们要认真贯彻十八届三中全会精神，抓住机遇、奋发有为，“逢山开路、遇水搭桥”，以改革破难题，以改革促发展，以改革赢红利，切实把改革创新贯穿于经济社会发展的各个领域、各个环节，努力推动经济更有效益、更高质量、更可持续发展和社会和谐稳定。

　　今年做好政府工作的总体思路是：全面贯彻落实中央、省和市委的各项决策部署，以全面深化改革为统揽，深入实施“蓝色珠海、科学崛起”战略，坚持稳中求进、提质增效，坚持深化改革、增强动力，坚持民生优先、富民惠民，促进经济稳中提速提质，促进产业加快转型升级，促进民生福祉持续改善，保持社会和谐稳定，加快建设生态文明新特区、科学发展示范市。

　　今年经济社会发展的主要预期指标是：地区生产总值增长10%，规模以上工业增加值增长12%，社会消费品零售总额增长13%，固定资产投资增长20%，外贸进出口增长8%，实际吸收外商直接投资增长8%，公共财政预算收入增长12%，城乡居民收入增长10%，居民消费价格涨幅和城镇登记失业率分别控制在3%和3.2%以内，节能减排完成省下达的任务。

　　今年政府工作的重点任务是：

　　（一）推进经济发展提速提质。

　　不断提升自主创新能力。加强创新平台建设，促进一批科研机构落户，新增一批工程中心和企业技术中心，加快建设中科院广州技术转移中心珠海中心、留学生创业孵化基地等项目。深入实施知识产权、标准化和品牌战略，推进“质量强市”和国家知识产权试点城市建设工作。探索建立知识产权交易机构，试行知识产权质押融资，支持探索设立科技创新银行。落实《人才开发促进条例》和“蓝色珠海高层次人才计划”，新引进和培育一批高素质人才。

　　保持投资合理较快增长。今年“十大重点工程”计划安排项目126项，总投资424.2亿元。强化项目建设服务保障，推动重点产业项目投产达效。协调推进格力商用空调、中海油精细化工园、3D打印特色园等一批重点项目。促成中海油天然气热电联产、碧辟三期、中国北车珠海基地等一批项目竣工投产。推动深水海洋工程装备制造基地一期、LNG接收站一期、佳能新工厂首期等一批新竣工项目投产达效，形成新的增长点。

　　积极扩大消费需求。发挥长隆国际海洋度假区首期开业的带动效应，促进旅游、餐饮等消费快速增长。促进信息、环保、文化、健康等新兴消费，发展养老服务、医疗保健、教育服务等新兴服务业。发展电子商务，鼓励商贸企业开展网上销售，建设电商园区和电商综合服务中心，吸引品牌电商服务商进驻，孵化本地服务商及网商品牌。推动华发商都、富华里、心海州等主城区商业综合体投入运营，加快金湾、斗门、高新、高栏等区域大型商业载体建设，新改造提升一批农贸市场。

　　稳定外贸进出口发展。发挥外贸促进政策综合效益，加快通关、结汇、退税速度，加大跨境贸易人民币结算、进出口保险支持力度，扶持企业扩大进出口。重点支持高新技术产品和一般贸易出口，扩大进口仓储、转口贸易。培育发展跨境贸易电子商务、市场采购贸易、外贸综合服务企业等外贸新业态。发展进出口企业总部和服务外包产业，加快建设保税区进出口商贸服务基地。加快加工贸易转型升级，促进企业向我市转移高附加值产品和研发、销售环节。支持企业“走出去”，开展海外并购和设立分支机构，扩大海外市场份额。

　　提高招商引资质量和效益。开展定向招商、产业链招商、以商引商。设立境外招商代表，推行驻点招商和代理招商。强化产业政策引导，鼓励各类资本资金更多投向“三高一特”产业领域。立足横琴优势推进总部企业招商，引进一批现代服务业和金融类企业，形成总部经济集聚效应。

　　促进各类企业竞相发展壮大。进一步落实和创新政策措施，培育大型骨干企业，发展非公有制经济，扶持中小微企业。动态实施大型骨干企业和重点民营企业培育目录，推动年主营业务收入超亿元企业成倍数递增。推进中小企业孵化器和创新产业化示范基地建设，重点帮扶成长型和科技型中小企业。鼓励银企开展战略合作，支持小额贷款公司、村镇银行等新型金融机构发展，设立政府注资的民营企业股权投资基金，为各类企业投融资提供更多扶持。扩大市场准入“平等待遇”，清理规范涉企收费项目，完善服务企业的“绿色通道”、“直通车”工作机制，充分发挥行业协会（商会）和工商类社会组织的引导服务作用，促进企业减负增效、发展壮大。

　　（二）增强“三大引擎”发展带动力。

　　实现横琴开发五年大变化。积极申报粤港澳自贸试验区，率先研究制定外商投资准入特别管理措施（负面清单）等创新制度，加快推动《横琴产业优惠目录》等一批新细化政策出台。加快推进已落户和在建的61个重点项目，争取开工建设横琴国际广告创意产业基地、丽新星艺文创天地等项目。建设粤港澳金融创新改革实验区，支持跨境人民币银团贷款、赴香港发行人民币债券等金融合作创新项目，推动设立和组建金融租赁公司、消费金融公司、合资全牌照证券公司等新型法人金融机构。促进多元化市场体系发展，争取横琴国际商品交易中心、稀贵金属交易中心尽快开业。完善基础配套，改造横琴大桥，推进横琴二桥、横琴第三通道等项目建设，建成横琴口岸过渡期通关设施主体工程。

　　推动高栏建设第三代港口。积极创建国家级生态工业示范园，申报高栏港综合保税区。深化西江战略，拓展内外贸航线，构建以多式联运为依托的港口供应链战略格局。推进15万吨级主航道改扩建、大型石化码头和粮食码头、10万吨级集装箱码头建设。加快发展海洋工程装备制造、清洁能源、现代化工和港口物流等产业集群，促成一批新项目开工建设、一批在建项目竣工投产。

　　建设国家一流高新区。推进高新技术产业和科技创新海岸加快发展。启动智能电网、医疗器械和高端电机制造专区建设，构建若干特色园区。建设科技金融创新试验区，健全科技金融产品创新和服务体系，推动科技型企业上市。实施孵化器倍增计划，建设火炬中心南方基地、创新广场、珠海信息港、清华科技园二期等项目。加快推进金山总部、前环总部基地等项目建设。

　　打造特色品牌园区。加快航空产业园发展，增强通航飞行服务站功能，完成通用机场建设前期工作，开工建设白龙河尾围填造地等工程，新引进一批重大航空项目，扩大航空产业规模。高水平办好第十届中国国际航空航天博览会，提升航展品牌效应。加大富山工业园招商和建设力度，着力在生物医药、环保产业、装备制造、高端新型电子信息、新材料等方面取得新突破。实施万山海岛开发五年总体方案，争取实现港澳游艇自由行，推进东澳玲玎海岸、桂山岛旅游集散枢纽、大小万山高端旅游区、万洋游艇俱乐部和游钓休闲渔业区建设，发展游艇、海钓等海岛海洋高端休闲旅游业。推进珠海国家农业科技园区、台湾农民创业园等园区建设，加快发展特色生态农业。

　　（三）推进交通建设“三年大会战”。

　　完善城市大交通骨干路网。突出抓好“三年大会战”交通工程的进度和质量，确保到2016年形成与港珠澳大桥紧密衔接的主骨架路网。配合国家和省加快建设港珠澳大桥主体工程及其连接线、西部沿海高速支线延长线等项目。继续推进港珠澳大桥珠海口岸工程、金琴高速等在建项目。开工建设香海大桥、洪鹤大桥、鹤州至高栏高速、南屏二桥、白石桥、有轨电车1号线二期等项目。建成疏港铁路专用线一期、梅华西立交、珠海大道金湾立交等项目。推进情侣路南段主辅线、斗门环城西路等一批城市干道改扩建工程。

　　完善海港、空港和口岸功能。启动珠海港总体规划修编，新建一批大型码头泊位，完成九洲港、香洲港货运码头功能搬迁工作。启动珠海机场总规修编，促进航空口岸开放，加快建设第二跑道。建设桂山港澳码头，推进珠澳跨境工业区专用口岸增加附属功能、拱北口岸联检楼二层通关大厅、万山港口岸新联检楼建设。

　　（四）提高城市宜居水平。

　　积极稳妥推进新型城镇化。实行城市总体规划、主体功能区规划、土地利用规划、产业发展规划、生态文明建设规划等多规融合、紧密衔接，进一步加强城市规划管理，坚守城市开发边界，提高土地节约集约利用水平。加快完善新型城镇化发展规划，优化城镇布局和形态。推进西部生态新城、平沙新城、航空新城、富山产业新城、唐家科教新城、珠海北站TOD的开发建设。加快建设市民文化广场，动工建设世邦国际商贸中心、梅溪商业综合体等项目，开展“三溪”搬迁安置区建设，推进香洲渔港、洪湾新渔港、城市之心、九洲商贸中心改造建设和拱北口岸地区综合开发项目。推进城中旧村改造。启动老旧小区综合整治和环境提升，深入开展市容市貌专项整治和“双限治乱”，提高城市综合治理水平。推进户籍制度改革，逐步建立城乡统一的户籍登记制度，分类有序地推进农业转移人口市民化。完善积分入户制度，逐步解决长期进城农民工及其家属落户问题。完善基本公共服务供给机制，健全异地务工人员子女教育、社保等配套政策，加快推动基本公共服务常住人口全覆盖。

　　创建珠海特色幸福村居。深化农村综合改革，推进农业经营方式创新。加强村居发展规划，提高现代农业发展水平，壮大集体经济，促进农民增收。搞好农村环境综合治理，促进村居净化、绿化、美化、亮化，争取两年完成209个村居污水处理设施建设。加快完成斗门北部四小联围海堤加固达标工程和农村水利设施建设。突出抓好村、社区“两委”换届选举工作，完善文化、体育、医疗、交通等公共服务设施。

　　加快智慧城市建设。完成国家智慧城市试点创建年度任务。推进4G网络建设和服务推广、光纤入户、WiFi热点覆盖，建设云计算中心。完善智能交通信息基础设施，抓好车联网、电子口岸等平台建设。提高产业创新发展的智慧支撑能力，推进公共创新、社会信用服务、智慧园区和旅游综合服务等智慧项目建设。优化城市智能管理，完善以市民卡、市民网页、网上办事大厅、12345城市服务热线为核心的智慧珠海综合服务体系，加快建设“数字城管”、食品药品安全监控、应急指挥调度等智慧项目。推进社会服务智慧化，完善智慧医疗、智慧社区等公共服务智慧平台。

　　推进生态文明建设。坚守生态底线，保护珠海良好生态优势，实现美丽与发展共赢，让广大市民永远呼吸到新鲜空气、享受青山绿水。大力创建全国文明城市和国家生态市，打赢“双创”硬仗。加强生态文明制度建设，公布实施生态控制线。建设林业重点生态工程，启动凤凰山森林公园、淇澳红树林湿地公园、横琴滨海湿地公园等项目，新建碳汇林2万亩、生态景观林带50公里。推进城市绿化景观档次提升工程，基本完成裸露山体整治复绿工作。推进广东1号绿道珠海延长段建设，新增绿道55公里。开展建筑楼宇低碳节能改造，制定绿色楼宇建筑标准在横琴试行。采取更有力有效的措施防污治污，加快推进全市污水管网投融资建设，开工建设前山污水处理厂新建一期，建成华新生活垃圾处理生态环保工厂，抓紧建设中信生态环保产业园首期、海岛污水处理系统、城区污泥无害化集中处置中心等项目。进一步完善城市生活垃圾收运体系。大力推进前山河流域环境综合整治提升。加快淘汰“黄标车”，建成机动车排气污染监控和防治工程。推进公交电动化，试行微公交运营，完善慢行系统，建成自行车主干道体系，建设公共自行车租赁系统二期工程。

　　（五）促进区域协同发展。

　　深化珠港澳更紧密合作。全面落实粤港、粤澳合作框架协议和CEPA补充协议，突出服务业和城市功能衔接，加强珠港澳旅游、会展、金融、物流、商务服务、文化创意、口岸和社会民生等领域合作，实现服务贸易自由化。完善珠港合作机制，成立珠港合作专责小组。深化珠港机场合作，扩大为粤港澳珠四方合作机制。推进粤澳新通道和港珠澳大桥人工岛口岸交通枢纽建设，探索“合作查验、一次放行”的通关模式。开展珠澳城轨对接首期工程。扩大建设粤澳合作产业园，承接更多优秀项目和投资落户。推动珠澳跨境工业区向珠澳跨境合作区转型升级。

　　扎实推进珠中江经济圈建设。实施珠三角五个一体化规划和珠中江合作计划，扎实推进29个年度重点合作项目。加快区域高速公路骨干网和连接线、市际快速通道、跨界公路等项目建设。探索共建产业一体化服务体系新模式，发布第二批珠中江共性技术与服务平台列表。完善区域环境管理一体化机制，共建珠中江优质生态环境。推进饮水联网工作，促进水资源基础设施共建共享。加强医疗卫生、社会保险、文化教育、社会治安等方面合作，推进基本公共服务对接。

　　支持对口帮扶地区加快发展。扎实推进对口凉山州扶贫协作项目，继续支援重庆市巫山县发展，深入开展帮扶阳江、茂名“双到”工作，加快珠海（阳江）产业转移园建设，抓好市内新一轮扶贫开发和革命老区村建设，完成省下达的促进粤东西北地区振兴发展的任务。

　　（六）大力推动改革创新。

　　探索对接国际化自由贸易规则。加快适应促进贸易投资自由化、便利化相关规则，推进法治化国际化营商环境建设。巩固商事制度改革成果，完善市场准入、运行、监管、退出制度，继续探索和创新强化事中、事后监管的措施。启动市场准入负面清单管理模式，探索对外商投资实行准入前国民待遇。加快研究制定港澳投资准入的特别管理措施，率先基本实现珠港澳投资便利化。消除各种隐性壁垒，落实“非禁即入”投资政策，制定市属项目公共资源竞争性配置操作细则，研究各类企业平等进入特许经营领域的方法，支持企业资本资金进入交通能源、社会民生、公共事业等领域。加快完善社会信用和市场监管两大体系。

　　深化财政金融体制改革。进一步改进预算管理，建立全口径编报体系。合理界定市、区事权，优化收入划分格局，合理配置支出责任。完善转移支付体系，扩大财政资金竞争性分配改革的范围和领域。建立重点税源企业迁移和企业总分机构汇总纳税涉及的税收利益分配机制，调整出口退税负担机制，落实结构性减税和收费减免政策，继续实施好“营改增”工作。深化投融资体制改革，开展政府投资项目公示试点。推广农村财务监管平台建设试点工作，成立农村养老基金互助社。发展股权投资、融资租赁、保理等新兴金融业态，支持企业利用多层次资本市场直接融资，探索设立小贷公司调剂中心和融资性担保再担保公司，鼓励设立社区金融服务点。完善资源性产品价格形成机制，推进水电气等价格改革。

　　加快国资国企改革创新。优化国有经济布局，提升国有资产配置效率，大力培育具有国际竞争力和影响力的国有企业和国有企业集团，增强国有经济活力、控制力和影响力。分类推进国有企业改革创新，推动竞争性国有企业产权多元化，推进公益性国有企业特许经营权改革。支持有条件的国有企业改组为国有资本投资管理公司。推进政府经营性国有资产集中统一监管。设立国有企业改革发展创新基金。推动国有企业完善现代企业制度。探索建立国有企业职业经理人制度。完善国有企业管理人员激励约束机制。

　　推进社会体制改革先行先试。制定社会建设规划纲要，加快创建全省社会建设法制化示范市。探索社区管理与服务新模式，推进城乡社区综合服务、村居民主自治向纵深发展，开展创建校园社区和企业社区试点工作。深化社会组织登记管理体制改革，进一步加大社会组织培育力度，强化社会组织能力建设。出台社会工作者登记管理办法，加强社会工作人才队伍建设。

　　深化行政体制改革。整合优化政府机构，进一步转变政府职能，理顺政府与市场的关系。深化行政审批制度改革，“减、放、转”一批审批事项，严格控制新设审批事项，公布保留的审批事项目录，实行动态管理和在线监控。试行政府部门权力清单。继续推进经济社会管理权限下放，加快实现管理重心下移基层。理顺食品药品监管体系，深化事业单位分类改革，继续推进事业单位法人治理结构试点。推进行政复议工作机制改革，探索行政复议网上办理模式。构建市、区、镇（街）、村（居）四级政务服务体系，拓展12345热线平台服务功能，加强电子政务、政府网站建设，推进政务公开，提升行政透明度和便民服务效能。强化法治思维，健全决策机制和程序，完善重大事项集体决策、专家咨询、社会公示和公开听证制度，提高科学民主依法决策的水平。认真落实市人大及其常委会的决议和决定，执行重大事项报告制度，主动接受人大监督。严格落实中央“八项规定”，开展群众路线教育实践活动，促进“转作风、提效能”。加强财政预算全口径监督，公开“三公”经费预算决算。加强审计与监察工作。积极预防腐败，严厉查处违法违纪案件，营造廉洁从政环境。

　　（七）着力改善和保障民生。

　　持续加大民生投入。坚持保障底线、突出重点、完善制度，切实做好民生工作，让发展成果更多更公平地惠及民生，让全市人民的好日子越过越红火。今年全市公共财政预算九项民生支出安排134.5亿元，占支出总额的58.2%，按可比口径计算，增长14.7%。

　　繁荣发展文体事业。深化文化体制改革，构建现代公共文化服务体系。加快“一院两馆”和基层文体设施建设。支持转制文化企业完善现代企业制度，扶持文化企业发展，推动文艺精品创作生产。办好国际马拉松、沙滩音乐节、公共艺术城市空间站等文体旅游活动。推进文体惠民工程，广泛开展群众性文体活动。加强历史文化资源挖掘和保护利用，深入创建历史文化名镇名村。

　　办好人民满意教育。积极创建省推进教育现代化先进市。深入实施“强师工程”。调整中小学免费教育政策，提高公用经费补助标准。发展公益普惠性学前教育，推进新一批镇中心幼儿园建设。加快推进斗门特殊教育学校、市技工学校、格力职业学院、UIC新校区等项目。抓好首批“粤教云”试点学校建设。组建职业教育集团。出台促进高等教育科学发展意见。加强校园和校车安全管理，创建“平安校园”。

　　提高医疗卫生服务能力。加快创建健康城市。成立市医管中心，推进公立医院改革。鼓励社会资本开办医疗机构、参与公立医院改制重组，逐步形成多元化办医格局。加快医疗卫生设施建设，建成市人民医院北区、市疾病预防控制中心等项目，推进市妇幼保健院旧区改造，加快市慢性病防治中心、市紧急医疗救援中心等项目前期工作。优化医疗资源配置，加强西部地区和海岛医疗卫生服务机构建设。基本完成区域医疗信息化项目。推动控烟立法。

　　促进更高质量稳定就业。落实促进就业各项政策，多渠道开发就业岗位，扎实做好离校未就业高校毕业生、城镇就业困难人员、农村劳动力等各类群体就业再就业工作。强化创业和技能培训，引导劳动力适应和促进企业转型升级。提升基层就业服务能力，完善基层劳动争议调解组织，探索企业职工欠薪保障制度和工伤预防长效机制，加快创建和谐劳动关系示范市。

　　健全社会保障体系。完善统一的城乡居民社会养老保险制度，扩大城乡居民养老和医疗保险覆盖面。完善基本医疗和生育保险制度，改革医疗保险支付制度，健全医疗保险与医疗救助即时结算制度。推进社会保险基金社会监督试点工作。健全社会养老服务体系，完善养老福利设施，推广社区居家养老服务。支持慈善事业发展，弘扬慈善感恩文化。完善残疾人社会保障和公平就业体系。

　　努力解决好住房问题。健全以公共租赁住房为主体的住房保障体系，规范保障性住房建设、审核、分配和管理。严守保障性住房储备用地不被挤占挪用。加快建设唐家人才公寓、南屏沁园等保障房项目。保持住房合理增长需求，促进房地产市场平稳健康发展。

　　构建平安和谐珠海。加快创建全国安全发展示范市，完善“一体系三平台”隐患排查治理体系，强化“一岗双责”和重点地区、行业和企业的安全监管责任，深入开展消防安全隐患整治，持续提升安全生产水平。加强社会治安综合治理，扎实推进“项目创安”工程建设，完善立体化社会治安防控体系，依法严密防范和惩治违法犯罪活动。创新群众工作方法，深入解决信访维稳问题。推进“六五”普法，加强人民调解、法律援助和社区矫正工作。坚持计划生育基本国策，加强综合服务管理平台建设，建设人口均衡型社会示范市。整合食品药品检验检测机构，加强食品药品安全监管，抓好市民“菜篮子”工程，提高农产品安全生产水平，创建国家食品药品安全示范市。认真做好双拥共建、人民防空、民族宗教、外事侨务、海防打私、全民禁毒、防震减灾等各项工作。

　　（八）办好十件民生实事。

　　一是扩大就业再就业。新增3家市级创业孵化基地，新增创业人数2600人；年内城镇新增就业45000人，失业人员再就业12000人，就业困难人员就业2000人。

　　二是继续提高民生保障水平。城乡月人均低保补差水平分别提高到400元和410元。农村五保年供养标准提高到11300元。城乡居民医疗保险财政补贴标准提高到340元。人均基本公共卫生服务经费标准提高到35元。为重度肢体残疾人提供上门居家康复服务，为残疾人家庭进行住宅无障碍改造。

　　三是促进教育公平。建成第二批10所镇中心幼儿园，新增4500个幼儿学位，建设一批新校区。城乡免费义务教育生均公用经费补助标准小学提高到950元、初中提高到1550元。免除中等职业教育一、二、三年级在校生中所有农村（含区、镇）学生、城市涉农专业学生和家庭经济困难学生的学费（艺术类相关表演专业学生除外）。

　　四是实施文化惠民工程。举办市民文化节和异地务工青年文化节，丰富市民文化生活。免费开放市文化馆新馆，新建60个社区文体公园和100个农村村居文化中心。

　　五是建设养老服务体系。建设1个养老服务示范培训基地，改造2—3所镇级养老机构，建设10—15个社区（村）居家养老服务站点，提高对民办养老机构的补贴标准，为全市养老机构提供社工巡回服务。

　　六是加强食品安全风险监测。重点做好校园食品安全科技工程项目，安装校园食品安全视频监控系统，实现校园食品风险测评（评估）、食品消费溯源，保障学生饮食安全。

　　七是发展绿色交通。完成有轨电车1号线首期工程，新投放500台纯电动公交车，增加出租车投放，新建第二期公共自行车租赁点，建设一批智能停车设施。

　　八是扩大安居工程覆盖面。启动老旧小区综合整治工程。新开工保障性住房2900套，基本建成2900套。推出公共租赁住房2300套。

　　九是让更多家庭用上天然气。实施天然气入户计划。新建市政燃气管道80公里，完成主城区现有12万户居民管道燃气用户天然气置换工作。

　　十是提升村居生产生活环境。推进209个村居垃圾和污水处理、农村电网改造、农村道路建设等项目，建设绿化、美化、亮化工程。

　　各位代表，新一年的发展蓝图已经绘就，“共建美丽城市、共享美好生活”令人向往、催人奋进。让我们在市委的坚强领导下，同心同德，开拓进取，为加快实现全市人民共同的城市理想而努力奋斗！
